# Melatonin inhibits bovine viral diarrhea virus replication by ER stress-mediated NF-κB signal pathway and autophagy in MDBK cells

**DOI:** 10.3389/fcimb.2024.1431836

**Published:** 2024-08-21

**Authors:** Yi-Qing Zhao, Xue-Fei Wang, Jia-Lu Zhang, Yi Wu, Jing Wang, Jiu-Feng Wang

**Affiliations:** ^1^ College of Veterinary Medicine, China Agricultural University, Beijing, China; ^2^ Key Laboratory of Applied Technology on Green-Eco-Healthy Animal Husbandry of Zhejiang Province, College of Animal Science and Technology & College of Veterinary Medicine, Zhejiang A&F University, Hangzhou, China; ^3^ Sanya Institute of China Agricultural University, Sanya, Hainan, China

**Keywords:** BVDV, melatonin, PERK-eIF2α-ATF4, NF-κB pathway, autophagy

## Abstract

Bovine viral diarrhea-mucosal disease (BVD-MD) is a contagious disease in cattle, caused by the bovine viral diarrhea virus (BVDV). This virus continues to spread globally, exerting pressure on both public health and the economy. Despite its impact, there are currently no effective drugs for treating BVDV. This study utilized Madin-Darby bovine kidney (MDBK) cells as a model to investigate the antiviral effects of melatonin against Bovine Viral Diarrhea Virus (BVDV) and its connection with endoplasmic reticulum (ER) stress. Our results show that melatonin can suppress BVDV proliferation in MDBK cells by modulating the endoplasmic reticulum (ER) stress-mediated NF-κB pathway and autophagy. Specifically, melatonin alleviated ER stress, inhibited the activation of IκBα and p65, regulated autophagy, and reduced the expression levels of pro-inflammatory cytokines. Further, when we treated BVDV-infected cells with the ER stress inducer thapsigargin, it led to significant activation of the NF-κB pathway and autophagy. Conversely, treating the cells with the ER stress inhibitor 4-phenylbutyric acid reversed these effects. These findings suggest that melatonin exerts its antiviral effects primarily through the PERK-eIF2α-ATF4 of ER stress-mediated NF-κB pathway and autophagy. Overall, our study underscores the potential of melatonin as an effective protective and therapeutic option against BVDV, offering insights into its anti-infective mechanisms.

## 1 Introduction

Bovine Viral Diarrhea Virus (BVDV) is a member of the *Pestivirus* genus within the *Flaviviridae* family (Al-Kubati et al., 2021) and is responsible for significant economic losses worldwide (Braun et al., 2019). Its genome consists of a single-stranded, positive-sense RNA with a large open reading frame (ORF) which is approximately 12.3 kb in length (Chi et al., 2022). This ORF encodes four structural and eight non-structural proteins (Ridpath, 2003). Clinically, BVDV manifests in symptoms such as mucosal necrosis, diarrhea, persistent infection, immunosuppression, and reproductive diseases (Braun et al., 2019). Since its first isolation in the US, BVDV has subsequently been reported in multiple countries including Kenya, Australia, Germany, Belgium, France, Canada, and China (Kalaycioglu, 2007). While classical live attenuated and inactivated BVDV vaccines have been in use for decades, their safety and efficacy remain subjects of debate (Griebel, 2015). Consequently, the investigation of antiviral drugs would be a critical strategy against BVDV infection.

Endoplasmic reticulum (ER) plays a vital role in cellular function (Almanza et al., 2019), triggering cascades of transcriptional responses that include autophagy, apoptosis, and inflammation, therefore maintaining its homeostasis is crucial for cells (Csordás et al., 2018). The ER stress response will be stimulated when plenty of viral proteins are synthesized in infected cells, also called the unfolded protein response (UPR) (Lewy et al., 2020). An emerging study has revealed that BVDV specifically activated the PERK-eIF2α-ATF4 branch of the UPR to facilitate its own infection (Wang et al., 2023). The NF-κB inflammatory pathway is commonly activated by many stimulants, such as ER stress and viral infection (Sun, 2017), further contributing to the expression of proinflammatory factors (Barnabei et al., 2021). It has demonstrated that swine fever virus (CSFV) infection triggered the NF-κB signaling pathway, preventing the virus from replicating (Zhai et al., 2021). In addition, hepatitis C virus (HCV) infection also promoted NF-κB pathway transport into the nucleus by regulating ER stress (Li et al., 2009). Autophagy is a self-regulatory program that maintains the normal operation of cells to adapt to external stimuli (pathogen infection, starvation, ER stress, etc.) (Levine and Kroemer, 2019). Previous studies have found that HCV regulated ER stress-mediated autophagy to facilitate its proliferation (Dash et al., 2016). Nevertheless, whether BVDV could cause ER stress-mediated NF-κB pathway and autophagy remains to be investigated.

Melatonin (MT) is an indole compound which is ubiquitous in nature and secreted by the mammalian pineal gland (Minich et al., 2022). It has a long shelf life with scalable production and can be easily transported without refrigeration (Pohanka, 2022). Recent studies demonstrated that MT has anti-inflammatory, antioxidant, relieving ER stress and regulating autophagy effects (Chitimus et al., 2020) and can be used as an effective strategy to prevent and control viral infections (Bonilla et al., 2004). Treatment with MT reduced the expressions of ER stress protein GRP78 and CHOP in rabbit hemorrhagic disease virus (RHDV) infection (Tuñón et al., 2013). Furthermore, our earlier research showed that the use of MT as the vaccine adjuvant increased the efficacy of Erns-LTB vaccine, including that MT could indirectly inhibit BVDV infection (Wang et al., 2021). Nevertheless, it is unknown whether MT prevents BVDV infection via influencing ER stress, NF-κB pathway, or autophagy in BVDV-infected cells. To address this gap, we investigated the role of MT as an effective antiviral agent against BVDV for the first time. Using an established Madin-Darby bovine kidney (MDBK) cell model, we explored the antiviral mechanisms of MT, focusing on ER stress, NF-κB pathway, and autophagy. Our findings offer new strategies for protection against BVDV invasion and infection.

## 2 Materials and methods

### 2.1 Cell culture and virus

MDBK cells were grown in DME/F-12 1:1 (Cytiva, Marlborough, MA, USA) containing 10% fetal bovine serum (Thermo Scientific, Waltham, MA, USA) and 1% penicillin/streptomycin (Thermo Scientific, Waltham, MA, USA) at 37°C in a cell incubator containing 5% CO_2_.

The BVDV 1-NADL strain with GenBank Accession no. M31182.1 used in this study was purchased from the Chinese Veterinary Culture Collection Center (Beijing, China).

### 2.2 Reagent

MT (purity > 99%) was from Solarbio (Beijing, China). 4-Phenylbutyric acid (4-PBA) (Zheng et al., 2016) was purchased from MedChemExpress (South Brunswick, NJ, USA). Thapsigargin (TG) (Zhang et al., 2014) was provided by Beyotime (Shanghai, China).

### 2.3 Virus titration

MDBK cells were seeded in 96-well plates and infected with BVDV at MOI = 1.0 serially diluted 8-fold when grown to 80%, each concentration was repeated 4 times, incubated at 37°C for 45 min, washed 3 times with PBS, contained 2% FBS DMEM/F-12 was added, and cultured in a 37°C cell incubator for 24 h. BVDV titers were calculated as TCID_50_ using the Reed-Muench method.

### 2.4 Western blot analysis

Cells to be harvested were treated with radioimmunoprecipitation assay (RIPA) lysis buffer (Beyotime, Shanghai, China) and 1 mM phenyl methyl sulfonyl fluoride (PMSF, Solarbio, Beijing, China) for 20 min at 4°C. Lysates containing cellular proteins were harvested and centrifuged at 13 000 g for 15 min, then supernatants were collected. Protein concentrations were detected with the BCA protein assay kit (Beyotime, Shanghai, China). After adjusting to a uniform concentration using 5 × SDS protein electrophoresis loading buffer (Beyotime, Shanghai, China) and RIPA lysis buffer, they were boiled for 10 min. Protein samples were added on 10-12% sodium dodecyl sulphate–polyacrylamide gel electrophoresis (SDS-PAGE) for electrophoresis and transferred to polyvinylidene difluoride (PVDF) membranes (Millipore Corp., Billerica, MA, USA). Blocking was performed with 5% skimmed milk for 2 h at room temperature followed by overnight at 4°C with the indicated primary antibody(BVDV E2, VMRD, 157; GRP78, Wanlei Bio, WL3357; PERK, Wanlei Bio, WL3378; eIF2α, Wanlei Bio, WL01909; phospho-eIF2α (p-eIF2α), Cell Signaling Technology, 3398; ATF4, Wanlei Bio, WL02330; CHOP, Abmart, T56694; p65, Cell Signaling Technology, 8242; phospho-p65 (p-p65), Cell Signaling Technology, 3033); IκB-α, Bioss, bs-1287R; phospho-IκB-α (p-IκB-α), Bioss, bs-18129R; LC3A/B, Cell Signaling Technology, 4108; p62, Cell Signaling Technology, 5114). Next, washed 3 times for 10 minutes each with PBST. The corresponding enzyme-labeled secondary antibody (Horseradish peroxidase-conjugated afnipure goat anti-mouse IgG (H+L), Proteintech Group, SA00001-1; goat anti-rabbit IgG (H+L), Proteintech Group; SA00001-2) was added and incubated at room temperature for 45 min and then washed 3 times with PBST for 10 min each. Observation was performed by ECL chemiluminescence kit (Beyotime, Shanghai, China). Finally, target proteins were quantitatively analyzed by ImageJ software.

### 2.5 RNA extraction and RT-qPCR

Total RNA from MDBK cells was extracted using RNAiso Plus (Takara, Kyoto, Japan) and then reverse transcribed into cDNA according to the instructions of the PrimeScript ™ RT reagent Kit (Takara, Kyoto, Japan). 2^−ΔΔCT^ method was used to calculate mRNA expression of related genes and GAPDH gene was applied as the standardized internal control. Primer sequences used in this experiment are shown in **Table 1**.

**Table 1:** RT-qPCR primer sequences used for this study.

Primers name	Direction ^a^	Sequence (5’ →3’)
BVDV 5’UTR	F	GAGTACAGGGTAGTCGTCAG
	R	CTCTGCAGCACCCTATCAGG
IL-1β	F	GGCAACCGTACCTGAACCCA
	R	CCACGATGACCGACACCACC
IL-6	F	GCTGAATCTTCCAAAAATGGAGG
	R	GCTTCAGGATCTGGATCAGTG
IL-8	F	GTTGCTCTCTTGGCAGCTTT
	R	CAGACCTCGTTTCCATTGGT
IL-18	F	TCAGATAATGCACCCCAGACC
	R	GATGGTTACGGCCAGACCTC
TNF-α	F	ACGGGCTTTACCTCATCTACTC
	R	GGCTCTTGATGGCAGACAGG
GAPDH	F	TTGTGATGGGCGTGAACC
	R	CCCTCCACGATGCCAAA

a F, forward; R, reverse.

### 2.6 Viricidal effect and viral life cycle assay

Viral inactivation assay: BVDV (MOI = 1) was incubated with 500 μM MT or DMSO for 2 h at 37°C. Then washed by PBS and repurified by ultracentrifugation at 90,000 g in 20% sucrose buffer (w/w). BVDV particles were resuspended in culture medium and then incubated in cells for 2 h at 37°C. Following PBS washes, MDBK cells were tested for viral infectivity with RT-qPCR.

Viral attachment assay: MDBK cells grown to 80% were pretreated with MT at a concentration of 500 μM or DMSO at 37°C for 1 h, followed by addition of BVDV (MOI = 1) for 2 h at 4°C. After washing 3 times by PBS, collected MDBK cell lysates were detected by RT-qPCR.

Viral internalization assay: To allowed BVDV virus attachment, BVDV at MOI = 1 infected MDBK cells for 1 h at 4°C. The unbound virus was washed off by 3 times with PBS (pH = 3). MDBK cells were then incubated at 37°C for 1 h in culture medium containing 500 μM MT or DMSO. Finally, RT-qPCR analysis was performed to detect MDBK cell lysates.

Viral replication assay: BVDV (MOI = 1) was added to 80% growth of MDBK cells at 37°C for 1 h. Non-internalized BVDV particles were removed by washing 3 times with PBS (pH = 3.0). Subsequently, MDBK cells were incubated with culture medium containing 500 μM MT or DMSO and placed in a 37°C cell incubator for 10 h. Finally, RT-qPCR analysis of harvested MDBK cell lysates was performed.

Viral release assay: BVDV (MOI = 1) was added to 80% growth of MDBK cells at 37°C for 1 h. PBS washed 3 times, then cells incubated in culture medium with 2%FBS at 37°C for 10 h. After washing 3 times with PBS, MDBK cells were incubated with culture medium containing 500 μM MT or DMSO and placed at 37°C for 2 h. The supernatants were collected for the determination of TCID_50_ (Yang et al., 2021).

### 2.7 Cell viability assay

MDBK cells were seeded for 2 × 10^4^ cells at per well in 96-well plates and allowed to grow for 24 h at 37°C. Subsequently, MT was added to MDBK cells at defined concentrations for 24 and 36 h, respectively. After reaching the exposure time, 10 µL CCK-8 was added to each well and placed at 37°C for 2 hours. According to the instructions, the absorbance of 96-well plate at 450 nm was detected by microplate reader and repeated for 3 times for cell viability analysis.

### 2.8 Immunofluorescence assay

MDBK cells were seeded in 24-well plates placed on glass slips (Solarbio, Beijing, China). Cells were covered with 4% PFA for 13 min, washed 3 times by PBS, incubated with 1% Triton X-100 for 13 min, washed 3 times by PBS, then blocking in 5% BSA for 1 h at room temperature to reduce nonspecific background. They were again washed 3 times by PBS and added with FITC-labeled goat rabbit/mouse IgG (Beyotime, Shanghai, China) for 1 h at room temperature. After washing 3 times with PBS, DAPI was added for 3 min and then washed by PBS, the washing solution was precooled with PBS throughout, and finally anti-fluorescence fading was covered on cell slides. Nikon A1 confocal laser scanning microscope was performed to take photomicrographs.

### 2.9 Inhibitor treatments

MDBK cells were pre-treated with 2 mM ER stress inhibitor 4-PBA and 2 µM ER stress activator TG for 37°C 2 h, challenged with BVDV infection (MOI = 1) for 37°C 45 min, and then kept in the culture medium of MDBK cells at specific concentrations (Wang et al., 2023).

### 2.10 Statistical analysis

Full text data were obtained from three independent replicate analyses and presented as mean ± SEM. The statistical analysis between groups was performes using GraphPad Prism 8.0 with one-way ANOVA or Student’s t-test. *P* < 0.05 was considered as statistically significant, * *P* < 0.05, ** *P* < 0.01, and *** *P* < 0.001.

## 3 Results

### 3.1 MT downregulated the replication level of BVDV

**Figure 1:**
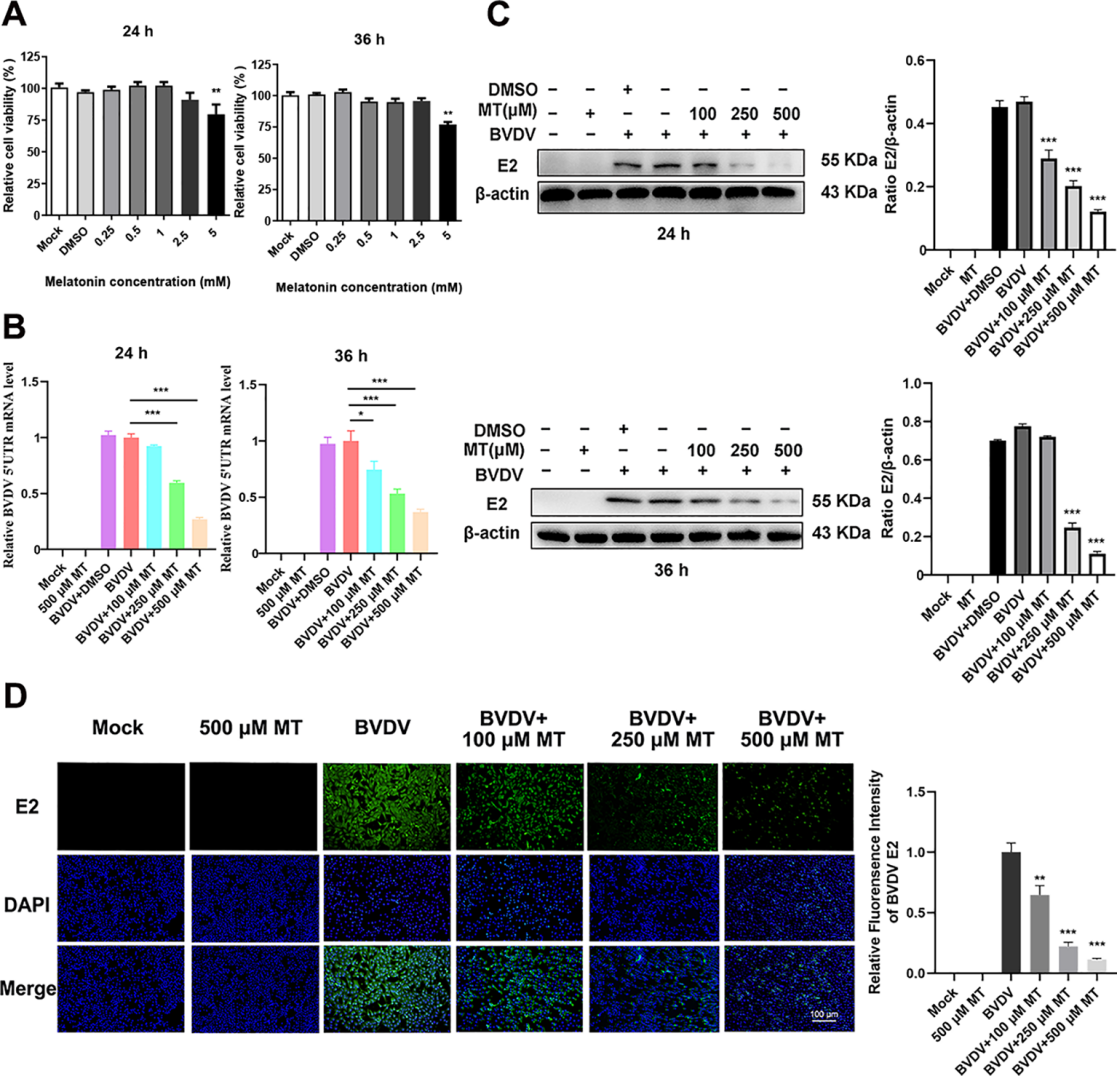
MT downregulated the replication level of BVDV. **(A)** Cytotoxicity of 0.25-5 mM MT in MDBK cells at 24 and 36 (h) **(B)** In BVDV-infected MDBK cells treated with 100, 250, or 500 μM MT, RT-qPCR was performed to measure the levels of BVDV mRNA expression at 24 and 36 (h) **(C)** The BVDV E2 protein expression levels at 24 and 36 h post-infection after treatment with 100, 250, or 500 μM MT. **(D)** The IFA image of BVDV E2 protein (green), DAPI (blue). The scale bar represents 100 μm. Data are presented as means ± SEM of 3 independent experiments. *, P < 0.05; **, P < 0.01; ***, P < 0.001.

The Cell Counting Kit 8 (CCK-8) assay was conducted to evaluate the cytotoxicity of various concentrations of MT (0.25, 0.5, 1, 2.5, 5 mM) in MDBK cells over 24 and 36 h. The results showed that MT concentrations of 0.25, 0.5, 1, and 2.5 mM did not significantly inhibit MDBK cell viability at either 24 or 36 h (**Figure 1A**). Based on these findings, concentrations of 100, 250, and 500 µM MT were selected for further experiments. To assess the inhibitory effect of MT on BVDV viral replication, MDBK cells were infected with BVDV for 45 min followed by 24 and 36 h with MT at concentrations of 100, 250, and 500 µM, respectively. RT-qPCR analysis of the BVDV 5’UTR mRNA level revealed a decrease in BVDV replication correlating with increased concentrations of MT at 24 and 36 h (**Figure 1B**). This was further corroborated by the analysis of BVDV E2 protein level, which showed that 250 and 500 μM MT significantly inhibited BVDV expression at 24 and 36 h, aligning with the RT-qPCR results (**Figure 1C**). The IFA demonstrated that MT treatment significantly reduced the number of BVDV-infected cells in a concentration-dependent manner, compared to both BVDV group and BVDV + DMSO group (**Figure 1D**). In summary, MT reduced BVDV E2 protein, decreased the number of BVDV-infected cells, and downregulated BVDV mRNA levels. These results reflected the activity of melatonin in inhibiting BVDV replication *in vitro*.

### 3.2 MT inhibited BVDV attachment and replication

**Figure 2:**
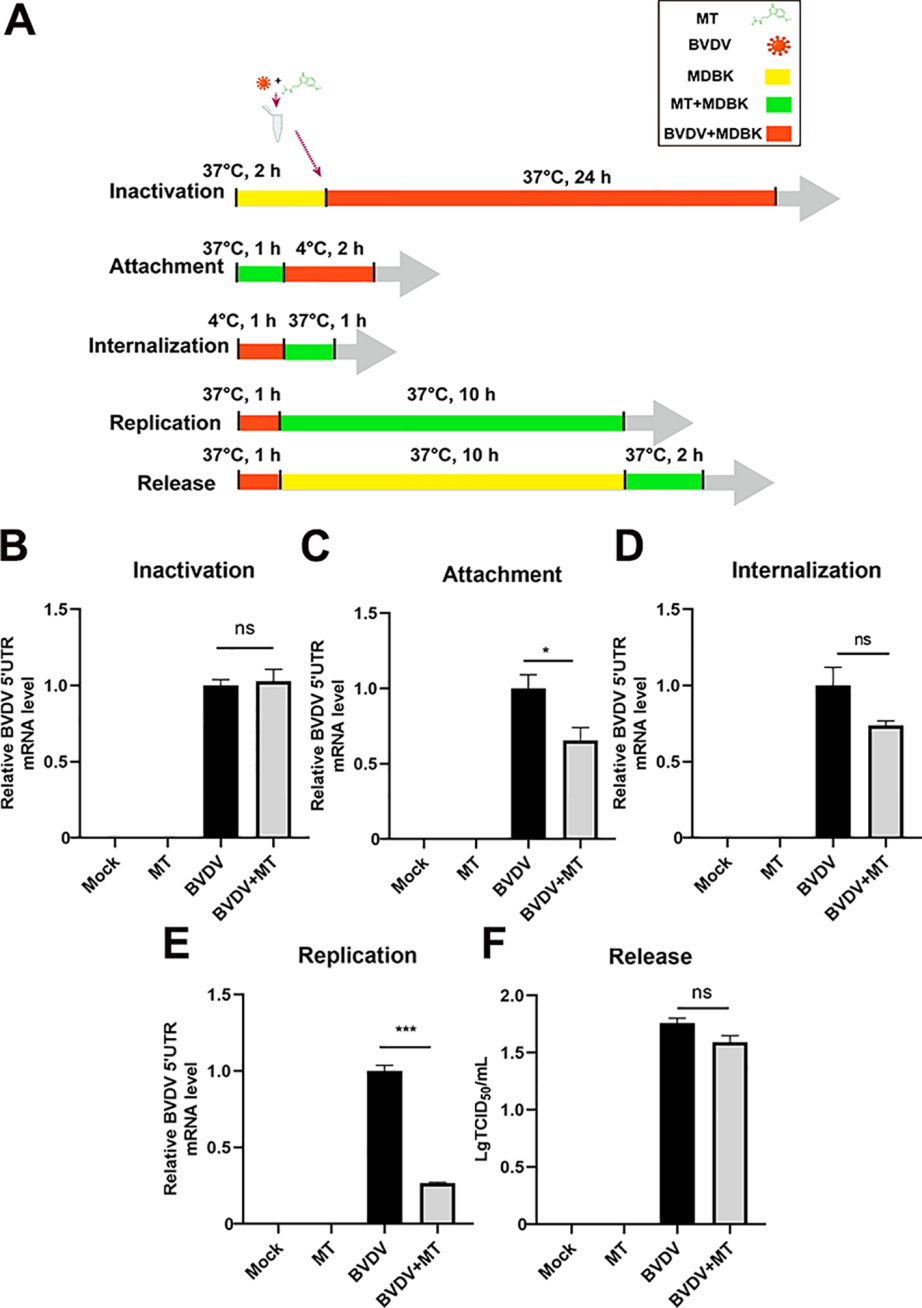
MT inhibited BVDV attachment and replication. **(A)** The schematic of DMBK cells treatment with 500 µM MT and BVDV infection in the inactivation, attachment, attachment, replication, and release processes. **(B)** RT-qPCR analyzed the viricidal effect of 500 μM MT on BVDV virions. The BVDV mRNA expression levels in MDBK cells with 500 μM MT treatment of BVDV replication cycle including attachment **(C)**, internalization **(D)**, replication **(E)**, and release **(F)**. Data are presented as means ± SEM of 3 independent experiments. *, P < 0.05; ***, P < 0.001; ns, not significant.

To further investigate whether MT could directly inactivate BVDV or influence specific stages of its replication cycle, we examined BVDV mRNA expression levels for inactivation, attachment, internalization, replication, and release by RT-qPCR (**Figure 2A**). Intriguingly, treatment with MT did not directly inactivate BVDV particles (**Figure 2B**). Next, we evaluated whether MT exerts its antiviral effects by affecting the BVDV life cycle including attachment, internalization, replication, and release (**Figures 2C-F**). The results of RT-qPCR revealed that MT treatment led to a downregulation of BVDV mRNA during the attachment and replication stages compared to the BVDV-infected group. However, MT had no significant impact on the internalization and release stages in BVDV-infected cells. Thus, MT inhibits the level of BVDV replication by affecting the attachment and replication phases of the BVDV life cycle.

### 3.3 MT suppressed the BVDV−induced PERK-eIF2α-ATF4 pathway of ER stress in MDBK cells.

**Figure 3:**
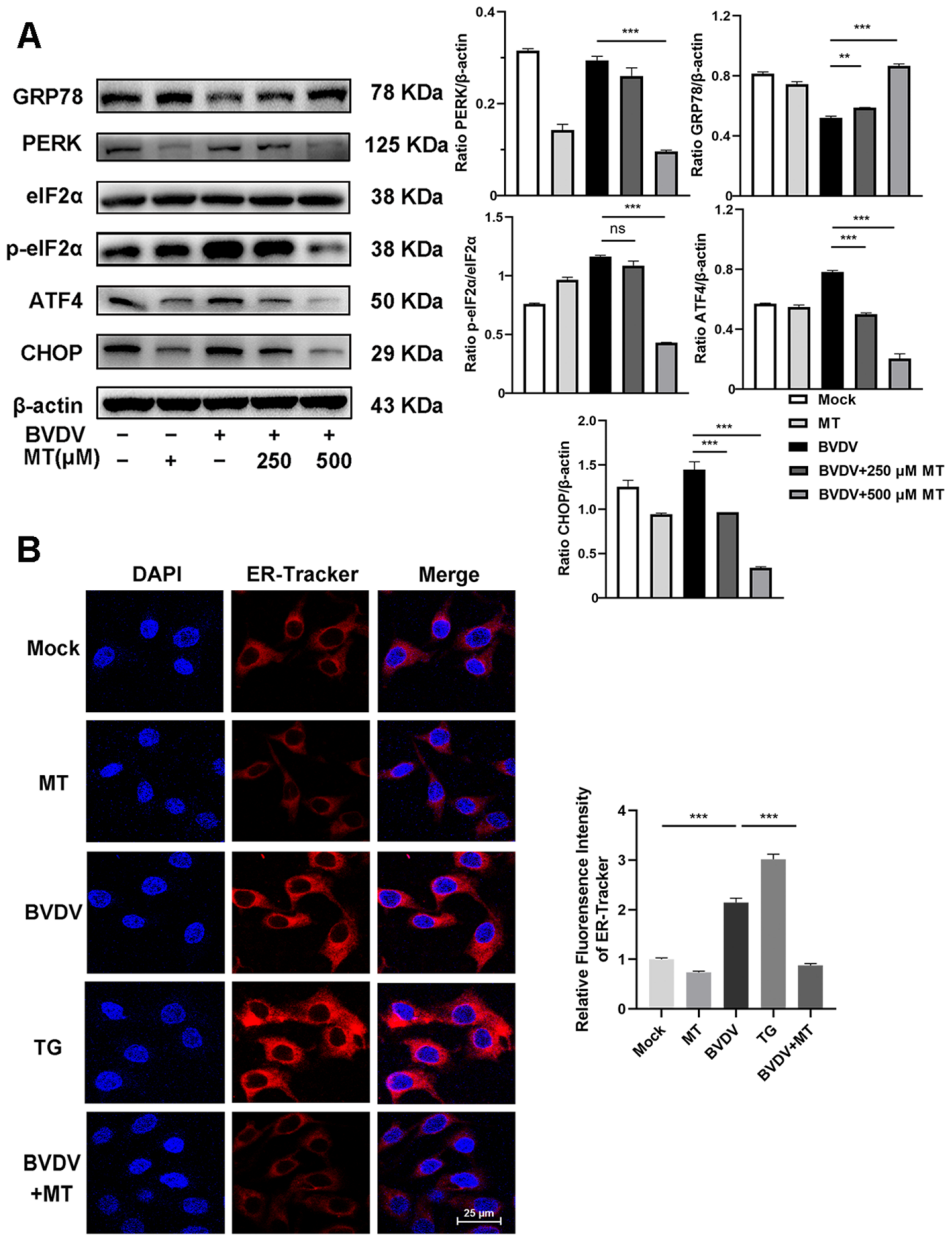
MT suppressed the BVDV−induced PERK-eIF2α-ATF4 pathway of ER stress in MDBK cells. **(A)** The expression levels of GRP78, PERK, p-elF2α, elF2α, ATF4, and CHOP protein were detected by western blot at 24 (h) **(B)** The IFA image of ER-tracker (red), and DAPI (blue). Scale bars represent 25 μm. Data are presented as means ± SEM of 3 independent experiments. **, *P* < 0.01; ***, P < 0.001; ns, not significant.

In this study, we investigated whether MT exerted its anti-BVDV activity through suppressing the PERK-eIF2α-ATF4 pathway of ER stress. Western blot analysis revealed that treatment with 250 and 500 μM MT downregulated PERK, p-eIf2α, ATF4, and CHOP protein expression levels and increased GRP78 protein level in BVDV-infected MDBK cells compared to the only BVDV infection group (**Figure 3A**). Additionally, IFA also demonstrated that MT treatment significantly reduced the fluorescence intensity of the ER tracker which represented ER stress levels in BVDV-infected MDBK cells (**Figure 3B**). Therefore, MT alleviated BVDV-induced activation of the PERK-eIF2α-ATF4 pathway of ER stress in MDBK cells.

### 3.4 MT regulated NF-κB signal pathway and autophagy in BVDV infection of MDBK cells

**Figure 4:**
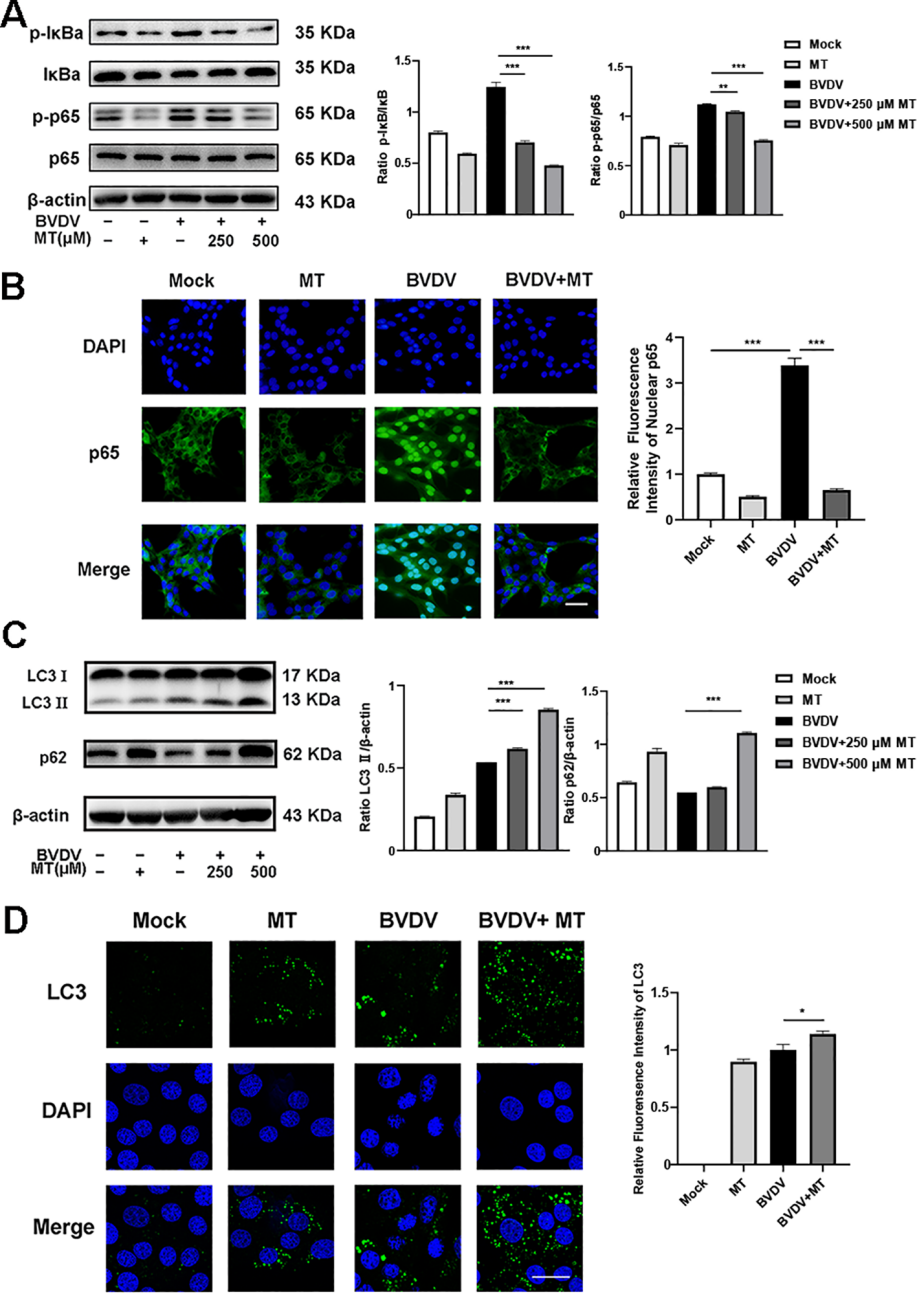
MT regulated autophagy in BVDV infection of MDBK cells. **(A)** p-p65, p-IκB, p65, IκB expression were analyzed by western blot. **(B)** p65 (green) immunofluorescence staining was used to observe p65 nuclear translocation. DAPI: blue, nucleus. Scale bar, 10 μm. **(C)** LC3 II, p62 expression were analyzed by western blot. **(D)** LC3 (green) immunofluorescence staining was used to observe autophagy. DAPI: blue, nucleus. Scale bar, 10 μm. Data are presented as means ± SEM of 3 independent experiments. *, *P* < 0.05,**, *P* < 0.01, ***, *P* < 0.001.

NF-κB signal pathway and autophagy are considered to play important roles after viral infection. To assess the effect of MT on NF-κB pathway and autophagy in BVDV-infected MDBK cells, the western blot was performed. The levels of p-IκB/IκB and p-p65/p65 were sharply elevated in BVDV-infected cells, and 500 μM MT treatment effectively suppressed activation of the NF-κB signaling pathway (**Figure 4A**). The expression and distribution of p65 detected by IFA were identical to the western blot findings (**Figure 4B**). Furthermore, the analysis indicated that 500 μM MT treatment upregulated the levels of autophagy marker proteins LC3 II and p62 compared to the group with only BVDV infection. This suggested MT involved in regulating BVDV-induced autophagy could potentially prevent the degradation of autophagy in MDBK cells (**Figure 4C**). Subsequently, the IFA results corroborated this hypothesis, showing that 500 μM MT treatment in BVDV-infected MDBK cells resulted in a higher fluorescence intensity of LC3 in MDBK cells than in the control or the BVDV-only infection group (**Figure 4D**). Therefore, MT could modulate the NF-κB signal pathway and autophagy in BVDV-infected MDBK cells.

### 3.5 The PERK-eIF2α-ATF4 pathway of ER stress participated in the mechanisms of MT against BVDV replication in MDBK cells

**Figure 5:**
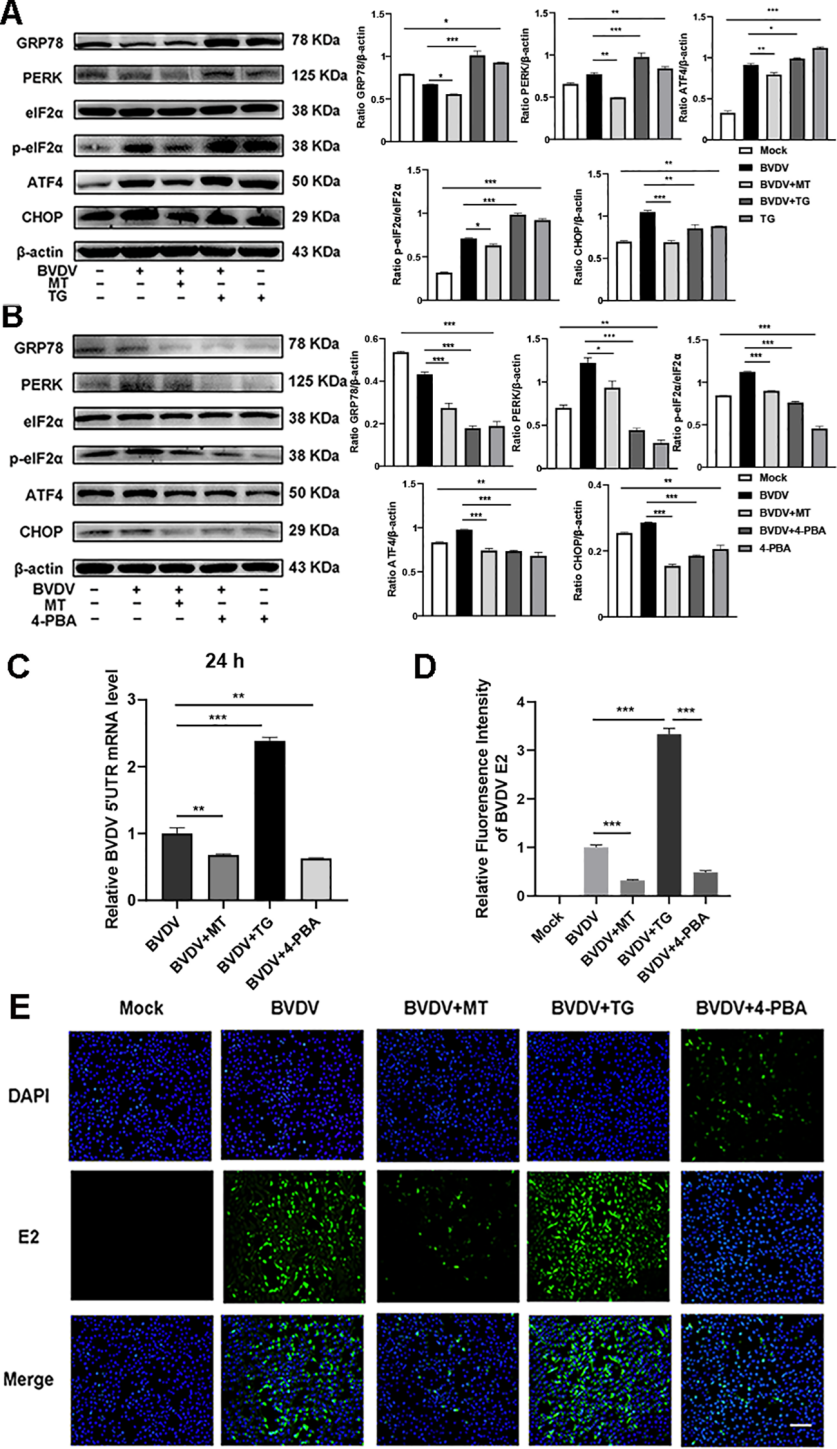
The PERK-eIF2α-ATF4 pathway of ER stress participated in the mechanisms of MT against BVDV replication in MDBK cells. Western blot detected the expressions of GRP78, PERK, p-elF2α, elF2α, ATF4, and CHOP protein in BVDV-infected MDBK cells with MT, TG **(A)**, or 4-PBA **(B)** treatment for 24 (h) **(C)** BVDV mRNA expression levels in MDBK cells with BVDV infection and treated with MT, TG, or 4-PBA for 24 (h) **(D, E)** The IFA image of BVDV E2 protein (green), DAPI (blue). The scale bar represents 50 μm. Data are presented as means ± SEM of 3 independent experiments. *, P < 0.05,**, P < 0.01, ***, P < 0.001; ns, not significant.

The above results indicated that BVDV infection activated the PERK-eIF2α-ATF4 pathway of ER stress. To delve deeper into the effect of ER stress in the inhibition of BVDV replication by MT, we applied Thapsigargin (TG, ER stress inducer) and 4-Phenylbutyric acid (4-PBA, ER stress inhibitor) in the subsequent experiments. TG treatment upregulated the expression of GRP78, PERK, p-eIf2α, ATF4, and CHOP proteins. However, this is reversed by 4-PBA and MT (**Figures 5A, B**). Besides, the results of RT-qPCR illustrated that TG-treated and BVDV-infected groups had higher expression of BVDV mRNA levels than the MT-treated and 4-PBA-treated groups (**Figure 5C**). IFA results further confirmed that TG treatment significantly increased BVDV-infected cells, whereas 4-PBA treatment reversed this effect (**Figures 5D, E**). Thus, the PERK-eIF2α-ATF4 pathway of ER stress is implicated in BVDV replication in MDBK cells. These results strongly illustrated that MT exerted its antiviral effect by modulating the BVDV-induced PERK-eIF2α-ATF4 pathway of ER stress in MDBK cells.

### 3.6 MT ameliorated PERK-eIF2α-ATF4 pathway of ER stress-mediated NF-κB signal pathway and autophagy in BVDV-infected MDBK cells

**Figure 6:**
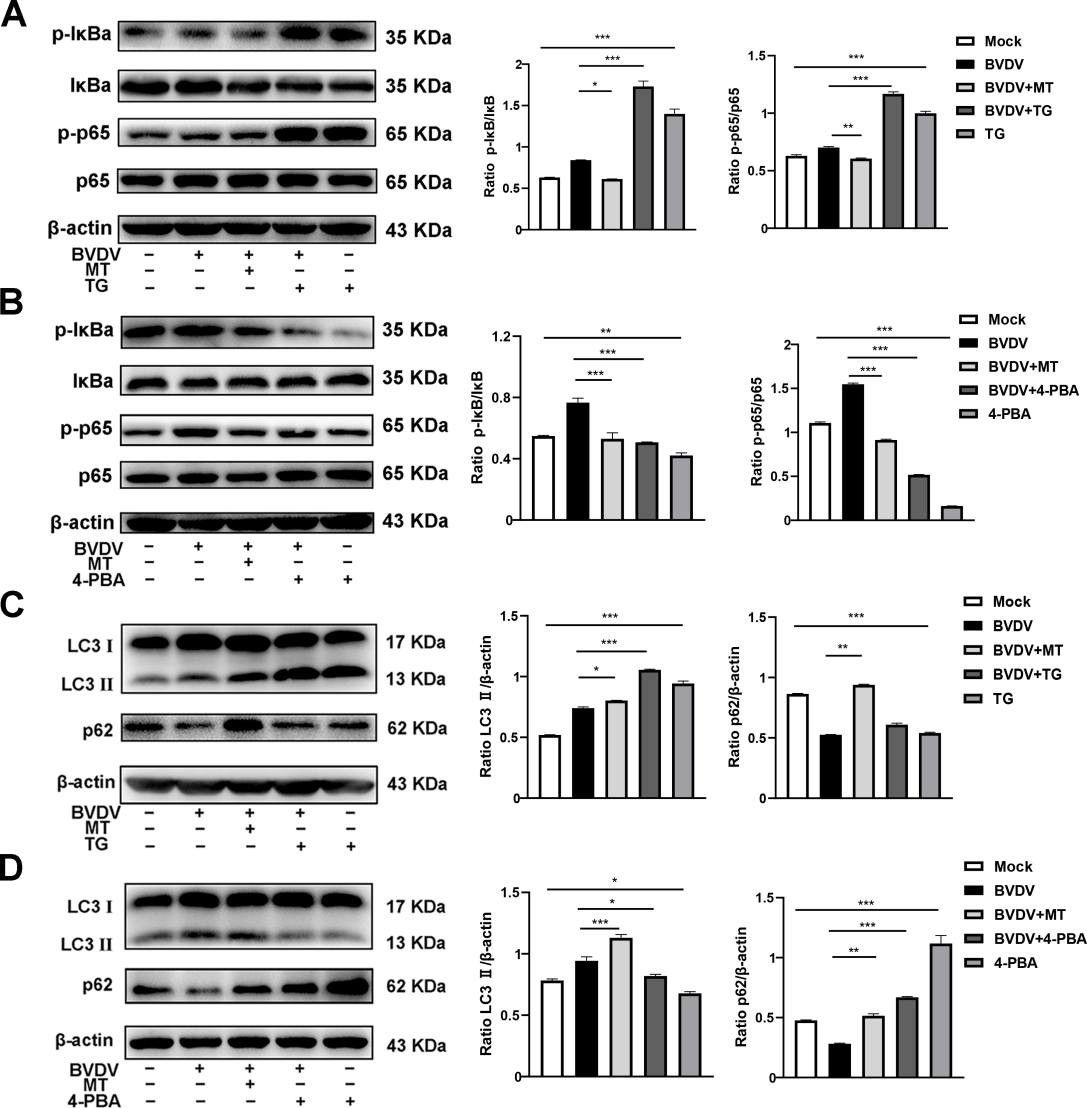
MT ameliorated PERK-eIF2α-ATF4 pathway of ERS mediated NF-κB signal pathway and autophagy in BVDV-infected MDBK cells. The expression levels of p-IκB, IκB, p-p65, and p65 protein detected by western blot in BVDV-infected MDBK cells with MT, TG **(A)**, or 4-PBA **(B)** treatment for 24 (h) The LC3 II and p62 protein expression levels in MDBK cells with BVDV infection and MT, TG **(C)**, or 4-PBA **(D)** treatment for 24 (h) Data are presented as means ± SEM of 3 independent experiments. *, P < 0.05,**, P < 0.01, ***, P < 0.001.

According to our present investigation, the molecular mechanism of MT against BVDV replication involves the PERK-eIF2α-ATF4 pathway of ER stress, which may be closely related to the NF-κB signaling pathway and autophagy (Lin et al., 2019; Li et al., 2022b). Hence, TG and 4-PBA were used to investigate impact of MT on PERK-eIF2α-ATF4 pathway of ER stress-mediated NF-κB signal pathway and autophagy. Compared to cells solely infected with BVDV, infected cells treated with TG had higher expressions of p-IκB/IκB and p-p65/p65, while 4-PBA treatment reversed this phenomenon (**Figures 6A, B**). In addition, both 4-PBA and MT treatment groups inhibited the BVDV-induced NF-κB signaling pathway (**Figure 6B**). Then, to further assess the effect of MT on PERK-eIF2α-ATF4 pathway during BVDV-induced autophagy, TG and 4-PBA were applied in additional experiments. TG treatment apparently upregulated LC3 II expression and downregulated p62 expression in BVDV infection cells (**Figure 6C**). 4-PBA treatment reversed these outcomes (**Figure 6D**) indicating the potential regulatory role for the PERK-eIF2α-ATF4 pathway of ER stress in autophagy. Additionally, MT could prevent autophagic degradation in BVDV-infected MDBK cells (**Figures 6C, D**). In summary, our results demonstrated that MT inhibited BVDV replication via modulating the BVDV-induced PERK-eIF2α-ATF4 pathway of ER stress-mediated NF-κB signal pathway and autophagy in MDBK cells.

### 3.7 MT alleviated BVDV-induced inflammation in MDBK cells

**Figure 7:**
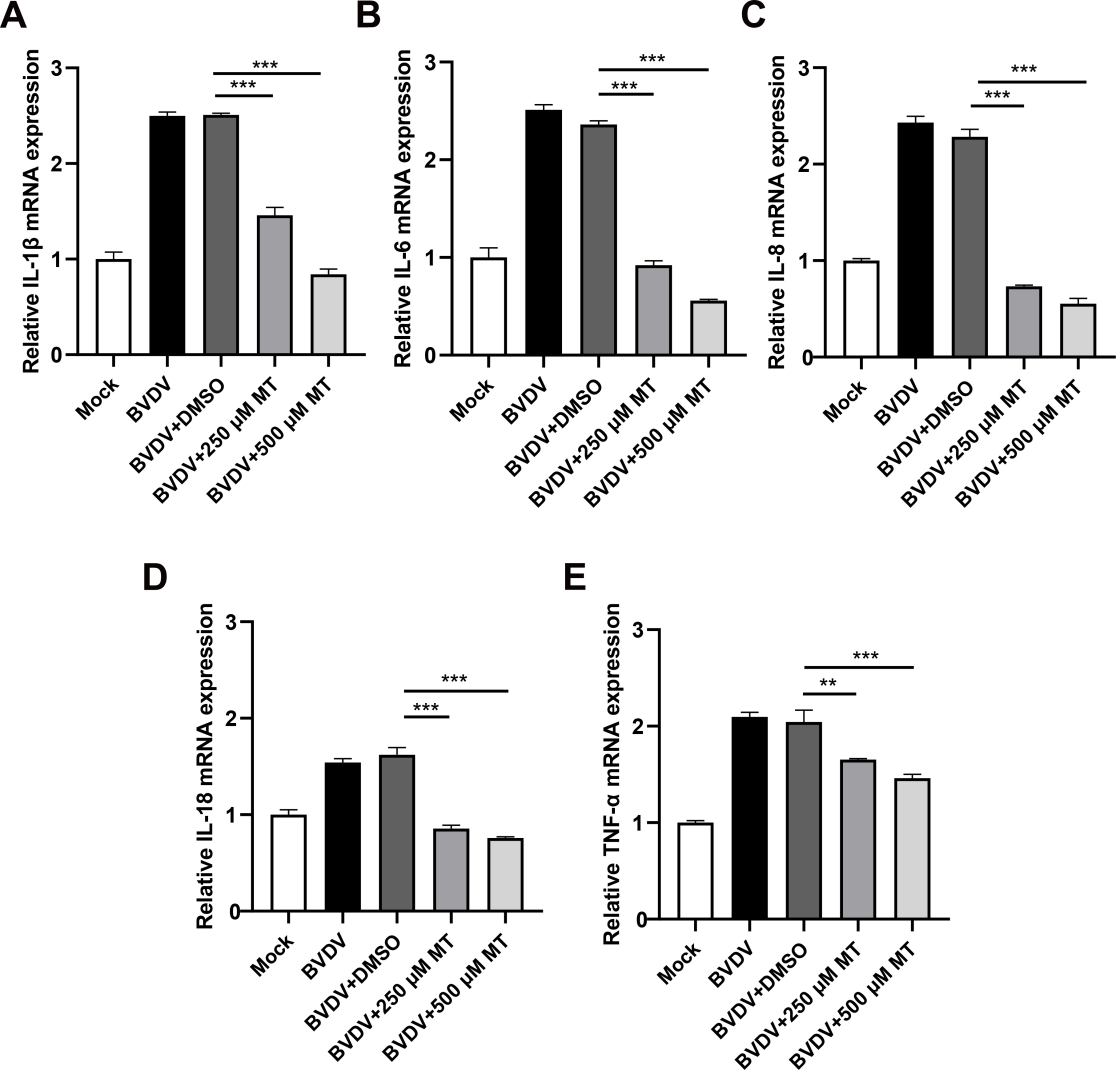
MT alleviated BVDV-induced inflammation in MDBK cells. The relative mRNA expressions of IL-1β **(A)**, IL-6 **(B)**, IL-8 **(C)**, IL-18 **(D)**, and TNF-α **(E)** in the BVDV-infected group, BVDV-infected group treated with MT (250, and 500 μM), or DMSO, and untreated group were detected by RT-qPCR at 24 h GAPDH gene mRNA level set as a standardized internal control. Data are presented as means ± SEM of 3 independent experiments. **, P < 0.01, ***, P < 0.001.

The activation of the NF-κB signaling pathway is closely linked to the release of pro-inflammatory cytokines (Napetschnig and Wu, 2013). To investigate whether MT treatment could reduce the levels of these cytokines during BVDV infection in MDBK cells, we conducted an RT-qPCR analysis. As anticipated, 250 and 500 μM MT-treated groups downregulated Interleukin-1β (IL-1β), IL-6, IL-8, IL-18, and tumor necrosis factor-α (TNF-α) caused by BVDV infection in MDBK cells (**Figures 7A-E**). These data suggest that MT is involved in regulating and attenuating the inflammatory and immune responses to BVDV infection by decreasing mRNA levels of pro-inflammatory cytokines in MDBK cells.

## 4 Discussion

Since the first report of BVDV, the virus has continued to spread globally, causing significant economic losses in the cattle industry (Braun et al., 2019). As a result, controlling the spread of BVDV and researching potent anti-BVDV drugs have become the top priorities. Although some promising natural compounds have been identified, the underlying mechanisms against BVDV remain unclear. Our study indicates that MT can inhibit BVDV replication by suppressing the PERK-eIF2α-ATF4 pathway of ER stress, downregulating the NF-κB signaling pathway, and modulating autophagy. This opens new avenues for controlling BVDV spread. To our knowledge, this study is the first report demonstrating that MT exerts the anti-BVDV effect via ameliorating the BVDV-induced NF-κB signaling pathway and autophagy through inhibiting the PERK-eIF2α-ATF4 pathway. To deeper elucidate the molecular mechanism of MT controls BVDV infection in MDBK cells, further studies are imperative.

MT has recently garnered attention as a potential antiviral agent, primarily because of its lack of side effects, low cost, and abundance. As a natural indole compound MT known for its diverse physiological functions (Reiter et al., 2014), including antiviral, anticancer, anti-inflammatory, and antioxidant effects (Chitimus et al., 2020). A Previous study found MT to be effective against Ebola virus infection (Anderson et al., 2015) and also acted as a main protease inhibitor for SARS-CoV-2 (Molina-Carballo et al., 2021), suggesting its potential as a preventive or therapeutic agent in controlling the COVID‐19 pandemic of humans (Anderson and Reiter, 2020). Simultaneously, MT has shown antiviral activity by directly affecting viral particles of swine coronaviruses *in vitro* (Zhai et al., 2021). These findings encouraged us to investigate the effect of MT on BVDV infection. In this study, we elucidated the molecular mechanism of MT resistance to BVDV infection of MDBK cells. It was found that melatonin acts mainly by affecting the attachment and replication phases of the BVDV viral life cycle, and its main mode of action may be through affecting the melatonin receptor or the cell surface receptor CD46 (Chen et al., 2021), but the specific mechanism of action remains to be explored.

ER is a vital organelle for viral modification and processing, therefore viral infection imbalances ER homeostasis and leads to ER stress (Lee et al., 2018). The PERK-eIF2α-ATF4 pathway is a crucial branch of ER stress that gets activated following viral infection (Lewy et al., 2020). In this study, the expressions of PERK, p-eIF2α, ATF4, and CHOP protein were apparently decreased with MT treatment in response to BVDV infection. These results demonstrated that MT could markedly alleviate BVDV-induced PERK-eIF2α-ATF4 pathway of ER stress in MDBK cells. In addition, we observed a close relationship between the PERK branch of ER stress and the NF-κB pathway in viral infections. A previous study indicated that the absence of eIF2α kinase could downregulate the NF-κB signaling pathway (Li et al., 2022a). PERK has also been shown to be essential for enhancing the transcriptional activity of NF-κB (Wang et al., 2022). We observed that MT administration significantly prevented the activation of the NF-κB pathway in BVDV-infected cells, which is consistent with its effect on PERK4-eIF2α-ATF branch of ER stress. Thus, we suspected that the function of MT in preventing BVDV replication is connected to the impact of ER stress on the NF-κB pathway.

Autophagy is a process that uses lysosomes to degrade self-damaged organelles and macromolecules (Mizushima and Komatsu, 2011). In comparison to the BVDV-infected group, our current data illustrated that MT-treated group raises the amounts of intracellular autophagy marker proteins p62 and LC3, which we hypothesized was because autophagy degradation was blocked. Besides, numerous studies showed that autophagy participates in ER stress and regulated under the guidance of various ER stress-related genes (Chipurupalli et al., 2021). The eIF2α/ATF4 pathway of ER stress could direct the transcriptional program of autophagy genes (Wang et al., 2023). Additionally, during MT therapy, autophagy was promoted in colorectal cancer (CRC) cells by ER stress (Chok et al., 2021). Studies demonstrated that HCV activated the eIF2α pathway of ER stress, then transcription factors ATF4 and CHOP were upregulated (Dash et al., 2016). LC3 II promoter can be activated by CHOP protein thereby upregulating transcription of autophagy gene LC3 II (Wang et al., 2014). Consequently, we presumed that ER stress could trigger autophagy in BVDV infection.

To further demonstrate the direct involvement of the PERK-eIF2α-ATF4 pathway of ER stress in BVDV-induced NF-κB signal pathway and autophagy, TG and 4-PBA were used for verification the role of PERK-eIF2α-ATF4 pathway of ER stress in BVDV infection as the typical inducer and inhibitor of ER stress *in vitro*. In this study, the inducer of ER stress TG caused a significant up-regulation of BVDV-induced PERK-eIF2α-ATF4 pathway, NF-κB signal pathway and autophagy. However, the inhibitor of ER stress 4-PBA reversed these effects. These findings strongly suggested that the PERK-eIF2α-ATF4 pathway of ER stress was critically participated in the BVDV-induced NF-κB pathway and autophagy. To date, the precise mechanism of NF-κB pathway mediated by ER stress during BVDV infection has not yet been elucidated by any study. Therefore, further investigation needs to be performed to understand how the ER plays an instrumental part and triggers a number of cellular signaling pathways and cascade reactions in BVDV infection.

**Figure 8:**
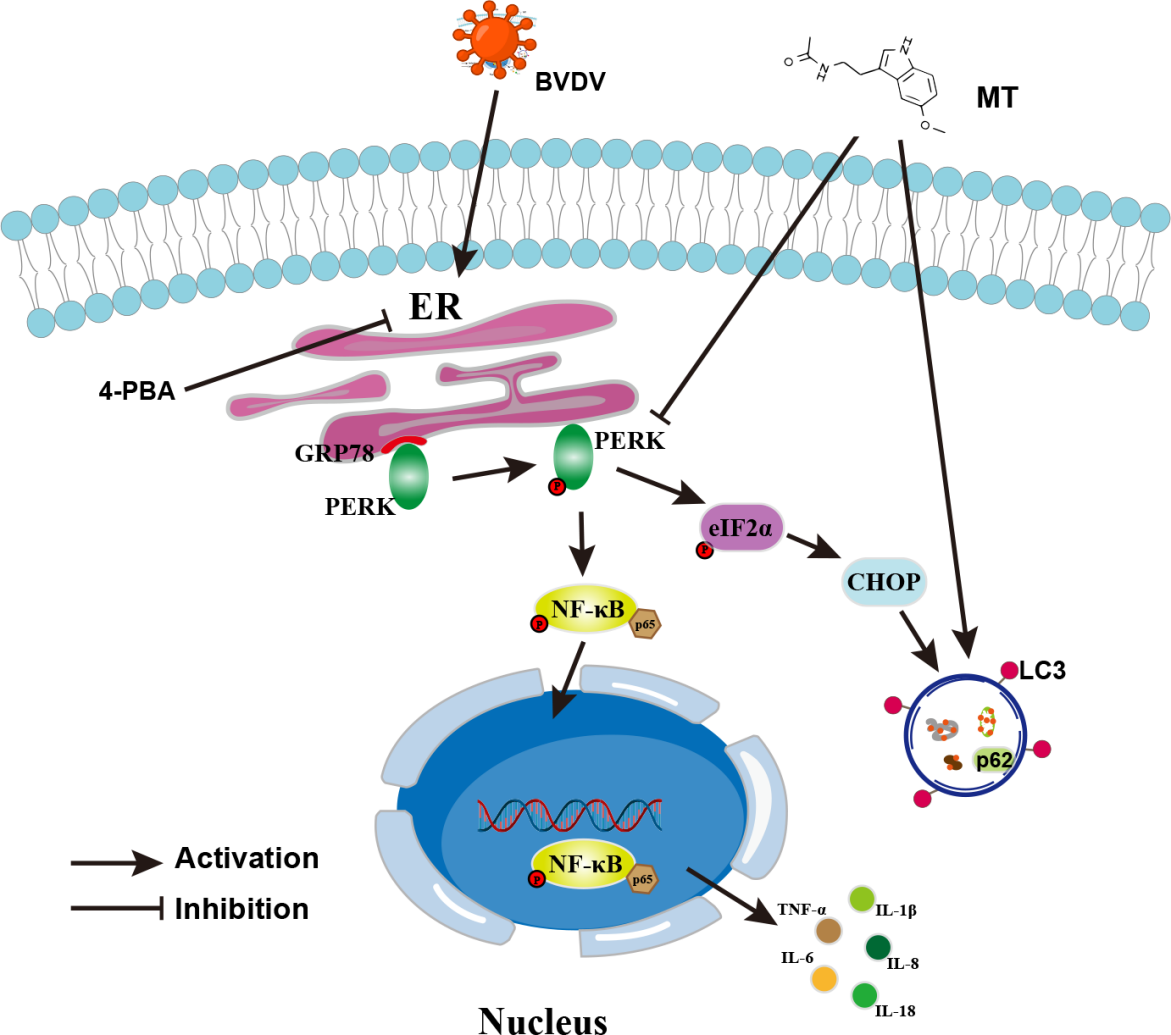
Schematic diagram of MT inhibiting BVDV replication via ER stress-mediated NF-κB pathway and autophagy. BVDV infection activates the PERK-eIF2α-ATF4 branch of the ER stress, promotes p65 phosphorylation and nuclear translocation, and regulates autophagy. MT exerts its anti-BVDV effect via ameliorating the BVDV-induced NF-κB signaling pathway and autophagy through inhibiting the PERK- eIF2α-ATF4 pathway.

In summary, this study was the first to demonstrate that MT suppressed the PERK-eIF2α-ATF4 pathway of ER stress, inhibited the NF-κB signal pathway, regulated autophagy, and reduced the expressions of pro-inflammatory cytokines in BVDV-infected MDBK cells. In addition, MT exerts antiviral activity via BVDV induced PERK-eIF2α-ATF4 pathway of ER stress-mediated NF-κB signal pathway and autophagy (**Figure 8**). This study deepens the understanding of the mechanism of BVDV infection in MDBK cells and provides a therapeutic reserve for controlling and treating BVDV.

## Data Availability

The original contributions presented in the study are included in the article/supplementary material. Further inquiries can be directed to the corresponding authors.
